# Determining the structure of functionalized graphene for tailored thermomechanical properties using ML techniques

**DOI:** 10.1039/d5ra07646c

**Published:** 2025-11-14

**Authors:** Ravil Ashirmametov, Alexandr Alpatov, Farrokh Yousefi, Narges Vafa, Siamac Fazli, Konstantinos Kostas

**Affiliations:** a Department of Mechanical and Aerospace Engineering, School of Engineering and Digital Sciences, Nazarbayev University Astana Kazakhstan ravil.ashirmametov@nu.edu.kz; b Department of Computer Science, School of Engineering and Digital Sciences, Nazarbayev University Astana Kazakhstan

## Abstract

Chemical functionalization of graphene with various chemical groups unlocks an infinite number of variations for nanosheet design modifications. However, the prohibitive cost of molecular dynamics simulations and the overwhelmingly large number of design variables render the inverse design problem intractable when conventional approaches are used. To this end, we develop an MD-powered, data-driven framework to enable fast and accurate identification of the layout that exhibits a given set of user-prescribed thermomechanical properties. Specifically, we generate a dataset with 1200 records, combining the layout and thermomechanical properties (Young's modulus, thermal conductivity, maximum stress and strain at maximum stress) of functionalized graphene sheets with hydrogen and methyl groups of appropriate coverages. A variety of regression models using Label and Bag-of-Words encoding were trained with Support Vector Regression, Ridge Regression and Gaussian Process Regression models showing best predictive performance, with considerably high values for the corresponding coefficients of determination (*R*^2^ > 0.9 for thermal conductivity, Young's modulus and maximum stress) on a hold-out test set, with mean absolute percentage error (MAPE) remaining below 1% in most cases. Finally, an evolutionary optimization process, in tandem with the trained Machine Learning (ML) models, was employed for finding graphene layouts that possess a set of user-defined target properties. MD-validations of the obtained designs confirmed the applicability of the approach while revealing acceptable deviations for thermal conductivity values and even better alignment for the mechanical properties. In summary, the proposed approach succeeds in a 7 orders of magnitude speedup in estimating the thermomechanical properties of functionalized graphene sheets when compared to pure MD simulations, and up to 6 orders of magnitude faster identification of layouts with prescribed properties, benchmarked on a nanosheet (220 × 100 Å) with 8528 atoms using a 64 core AMD EPYC workstation.

## Abstract 2

Developed framework enables the inverse design of graphene sheets for finding layouts possessing a predefined set of thermomechanical properties. Systematic analysis of 600 unique graphene layouts using MD to map trends in target properties.

## Graphical abstract

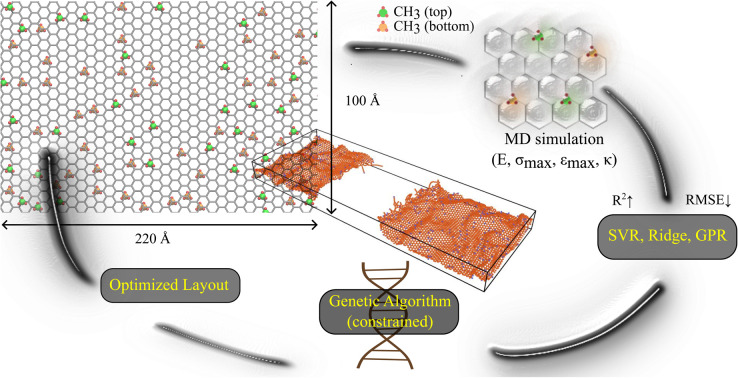

## 1 Introduction

Graphene has emerged as a “material of the future” due to its exceptional thermal transport capabilities, mechanical stiffness, electric conductivities and optical properties.^1–3^ However, for several applications, these exceptional properties of pristine graphene may not perfectly align with their specific requirements. Hence, for many practical cases, controlled modification of graphene sheets is required for matching the material to the application at hand.

A variety of strategies have been explored over the years for modifying the structure and properties of graphene with intentional lattice imperfections (vacancies, interstitial atoms, Stone–Wales defects and dislocations), as well as extrinsic defects, such as chemical functionalization, doping, and others.^4^ Chemical functionalization has been extensively studied in the pertinent literature and constitutes one of the commonly pursued approaches in graphene engineering. By covalent attachment of various functional groups, such as hydrogen, methyl, hydroxyl and other more complex molecules, a carbon grid can be locally or fully transformed to an sp^3^ hybridized sheet.^5^ This transition leads to significant changes in mechanical, electric and thermal properties and general behavior of the resulting nanosheet. Thus, controlled functionalization is a powerful mechanism, enabling delicate tuning of graphene properties to match the specifications for a wide range of practical application, including polymer nanocomposites, biosensors, solar cells, and drug delivery systems among others. In this study, we focus on hydrogen and methyl functionalization of graphene, which generally leads to a degradation of thermomechanical properties in the resulting graphene-based sheet.^6–8^ Simultaneously, other properties, such as electrical properties, *e.g.*, conductivity, carrier mobility, and bandgap, are also modified.^9^ These modifications are beneficial for specific applications with such needs, and thus, functionalized graphene finds numerous applications in nanocomposites,^10^ semiconductors,^11,12^ gas separation,^13^ and water treatment.^14^ Recent studies have also investigated quantitative linking of graphene-based materials to thermal and mechanical properties *via* heterostructures. These research studies highlight extensive efforts directed toward the employment of process–structure–property relationships in graphene-related materials, such as peptide–graphene sensors,^15^ heterogeneous composites,^16^ and composite fibers.^17^ As reported in pertinent literature,^12,18–20^ even relatively small percentages of functionalization (up to 20%) can significantly reduce thermal conductivity, electrical conductivity, tensile stiffness, maximum stress, and fracture strain at room temperature, compared to pristine graphene. This degradation has been observed in both experimental and theoretical studies using MD simulations. Understanding and quantifying the behavior of these effects is an essential enabler for the design and tuning of graphene-based materials for targeted engineering applications.

Molecular dynamics (MD) simulations constitute a valuable tool in exploring structure–property relations for nanomaterials at the atomic scale. MD permits direct calculation of mechanical, thermal, and transport properties under precisely controlled conditions commonly inaccessible experimentally.^21,22^ MD provides critical insight into how chemical variations, distributions of defects, and hybridization variations influence bond strength, phonon transport, and failure modes^6–8,23^ for functionalized graphene and other graphene-based nanosheets. Due to MD's atomistic-scale computations, the estimation of Young's modulus, tensile strength, fracture strain, and thermal conductivity value for graphene-based sheets has been repeatedly shown to be sufficiently accurate.^24–26^ However, this accuracy comes with a significant computational burden that renders identification of appropriate graphene-based layouts practically intractable in large design spaces. Therefore, coupling of MD simulations with data-driven models becomes a necessity in facilitating practical inverse design computations.^27,28^

Various researchers^29,30^ have already reported the successful use of Machine Learning (ML) models in predicting the physical properties of relevant materials with extremely high precision, as for example, the prediction of thermal conductivity^27^ with a coefficient of determination (*R*^2^) exceeding 0.95. Furthermore, high-predictive accuracy coupled with negligible inference cost render the usage of ML models in inverse design of structures with application-specific properties^28,31^ a feasible endeavor. However, the use of ML models in the inverse design of graphene-based sheets is still largely underexplored. The functionalization percentage, the location of the functional groups, as well as the type of molecular bonds are among the most important parameters in estimating the physical properties of functionalized graphene nanosheets. Therefore, one of the prerequisites for a successful employment and training of relevant ML models is the selection of an appropriate encoding of these parameters for a given functionalized graphene sheet.

Transforming the nanosheet layout, *i.e.*, the position and connectivity of atoms and molecules, into an appropriate representation for ML modeling can be challenging as various objectives need to be satisfied at the same time, *i.e.*, low-dimensional, parsable, numeric (vectorial, matrix, graph, *etc.*) representation, which can accurately capture all features of the nanosheet's layout and its relation to the physical properties of interest; see Xu *et al.*^32^ for a comprehensive review of challenges and perspectives in ML for energy chemistry. Functionalized graphene sheets pose additional challenges in comparison to regular molecules. For example, the number of atoms and molecules may vary significantly, presenting challenges for advanced and memory-inefficient encoding strategies. Furthermore, functional groups' placement cannot be entirely determined in a purely 2D encoding. The most common methods of molecular representation, as described by Raghunathan and Priyakumar^33^ and Xu *et al.*,^32^ include SMILES strings, molecular fingerprinting algorithms, Coulomb Matrices and their variants, and graph representations. Further processing of such representations, including extracting word embeddings, distance-based weighting, and Bag-of-Bonds encoding, is also applied to some of these representations when used in ML frameworks.

Molecular graph encoding is a promising State-of-the-Art method of molecular representation that preserves the spatial structure. In this approach, a molecule is transformed into its graph representation *G* = (*V*, *E*), where *V* is the set of vertices and *E* is the set of edges. The elements of *V* correspond to individual atoms, while *E* = {(*v*_1_, *v*_2_)∣*v*_1_, *v*_2_ ∈ *V*} consists of pairs representing the chemical bonds between atoms. This representation naturally supports the use of graph-based Machine Learning methods, such as graph neural networks (GNNs), which have shown significant promise in tasks like property prediction and molecular classification.^34^ However, this encoding technique scales heavily with the complexity of the ML model used with them and, as such, requires a large and extensive dataset.

An alternative approach to molecular encoding would be to extract word embeddings from SMILES strings. Recent research has shown significant progress in this area, with pre-trained vector similarity-based embedding models like Mol2Vec,^35^ as well as transformer-based models like MOLBert,^36^ achieving remarkable results on different Machine Learning tasks. However, these methods are not well-suited for encoding graphene-based sheets used in this work, as they require large numbers of regular molecular data without the relative homogeneity exhibited in graphene-based nanosheets.

A simpler and more intuitive approach for converting 2D molecular structures into numerical vectors for Machine Learning applications is Label Encoding. Label encoding operates by constructing a dictionary of all ASCII characters in a SMILES string of the molecule and replacing each character with a corresponding numerical value. This allows for interpretability, as the original molecule can be easily reconstructed by applying an inverse dictionary, and encodes some spatial relations between the atoms in a molecule. However, it does not reduce the dimensionality of the data and assumes that every molecule is of fixed length. For the dataset described in this paper, this leads to a set of 8528-feature vectors over 300 samples, which, according to Ying^37^ poses risks of overfitting. Another rather simple but efficient way to extract features from graphene-based sheets would be to consider the present molecular substructures, such as atoms, doping, or functionalization, and use the frequencies as features in an approach known as “Count Vectorizing”, “Bag-of-Words”, or “Bag-of-Features” encoding.^38^ This allows a significant reduction in the number of features.

Despite significant progress in analysis of the effect of certain defects or functionalization on thermomechanical properties of graphene, there is still some gap in fully understanding (a) the effects of functional groups' placement for the same coverage levels, (b) the predictive performance of ML regression models that account for such effects, and (c) the appropriate encoding schemes for large homogeneous graphene sheets. Finally, solving the inverse-design problem in the context of graphene-based layouts is being actively pursued in pertinent literature.

The major objective in this study is to develop an ML-powered inverse-design optimization framework that will allow for a relatively accurate and efficient prediction of the layout for a given set of thermomechanical properties. In this work, we limit our scope to hydrogen and methyl functionalization of graphene nanosheets and we demonstrate that the suggested approach is feasible and permits its further development and extension to support the engineering design of nanosheets with specified properties. In summary, we consider that this work significantly contributes to the pertinent literature by

• Systematically evaluating the impact of hydrogen/methyl functionalization on graphene sheets' thermomechanical properties,

• Identifying relevant ML-based regression models with high predictive performance,

• Enabling the cost-effective inverse design of functionalized graphene sheet layouts with tailored thermomechanical properties, and

• Highlighting areas where additional studies can be focused to improve and/or extend the obtained results.

The remainder of the document is structured as follows: Section 2 presents the functionalized graphene sheets targeted in this work, discusses the computational approach used for estimating their properties using MD simulations, as well as the selection of ML-regression models and their training. Section 3 showcases the most important results and observations from this work, while Section 4 summarizes our findings and proposes the next steps in the development and future extensions of the presented framework.

## 2 Methodology

This work begins with the preparation of a large number of valid functionalized graphene nanosheets, which are then processed *via* MD simulations to extract their relevant thermomechanical properties. The input and output results are compiled into datasets with Bag-of-Words and label encodings for the nanosheet layout. These datasets are used in training a variety of regression models, and their property-prediction performance is systematically compared in sequel. The most promising models are then employed in the identification of nanosheet layouts for given sets of thermomechanical properties by solving the corresponding optimization problems. Finally, the obtained optimal layouts are verified *via* MD simulations. The relevant details of each step are discussed in the following subsections with the obtained results being presented in Section 3.

### 2.1 Nanosheet preparation and assessment

**Fig. 1:**
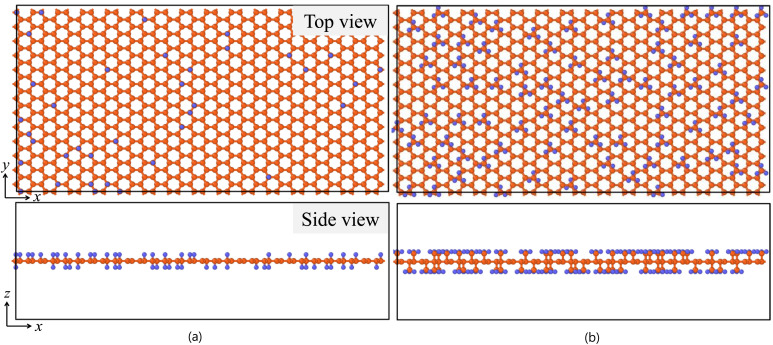
Schematic representation of (a) hydrogen- and (b) methyl-functionalized graphene. The upper and lower panels correspond to top and side views, respectively. Carbon atoms and hydrogen atoms are depicted with orange and blue spheres, respectively; only a small part of the complete sheet is shown.

For the compilation of the datasets, a base graphene nanosheet with 8528 carbon atoms was firstly constructed while having dimensions of 220 × 100 Å along the *x* and *y* directions, respectively; see also Fig. 1. Dimensions and size were selected to ensure bulk-like behavior under periodic boundary conditions, minimize artificial periodic interactions, and yield reliable thermomechanical property predictions. Subsequently, 600 unique and valid functionalized graphene sheets were generated using a python script which randomly placed hydrogen or methyl functional groups while ensuring functionalization within the preselected percentage ranges for each functional group, and the validity of the resulting nanosheet. The selected functionalization percentages ranged from 0 to 15% for hydrogen and up to 12% for methyl with no mixed functionalization. Furthermore, to avoid steric overlap, due to methyl's larger size, a minimum spacing of 3.5 Å between adjacent methylated sites was maintained, which is the main reason that we did not went up 15% methyl functionalization as it was hard to generate sufficiently different layouts with high functionalization percentages in this case. For non-zero functionalized cases, 20 to 25 unique layouts were generated for similar percentage levels. Small indicative regions from one hydrogenated and one methylated graphene nanosheet are shown in Fig. 1.

Non-equilibrium molecular dynamics simulations (NEMD) were conducted using the open-source Large-Scale Atomic/Molecular Simulator (LAMMPS)^21^ software package to determine the thermomechanical properties of the prepared functionalized graphene sheets. Periodic boundary conditions were applied for in-plane directions (*x*, *y*), and the free boundary condition, with a minimum height of 20 Å, for the out-plane (*z*) direction. For modeling atom interaction, we employed the adaptive intermolecular reactive bond order (AIREBO)^39^ model with a cutoff scaling factor of 2.5,^23^ which is a computational interatomic potential used in similar studies. This potential features repulsive and attractive pair interaction functions that fit bond properties, single-bond torsional, and long-range atomic interactions. The system was initially relaxed using the conjugate gradient algorithm to minimize its energy and eliminate nonphysical initial configurations. To achieve thermodynamic relaxation and release residual stresses, the entire system was equilibrated in the isothermal–isobaric (NPT) ensemble at room temperature and zero pressure for 1 ns using the Nosé–Hoover thermostat and barostat. Subsequent equilibration was performed using the canonical (NVT) ensemble for an additional 1 ns to ensure full thermodynamic equilibrium. Following equilibration, the production runs were performed to calculate the thermomechanical properties with a timestep of 0.1 fs being used for integrating the motion equation with the velocity Verlet algorithm.

**Fig. 2:**
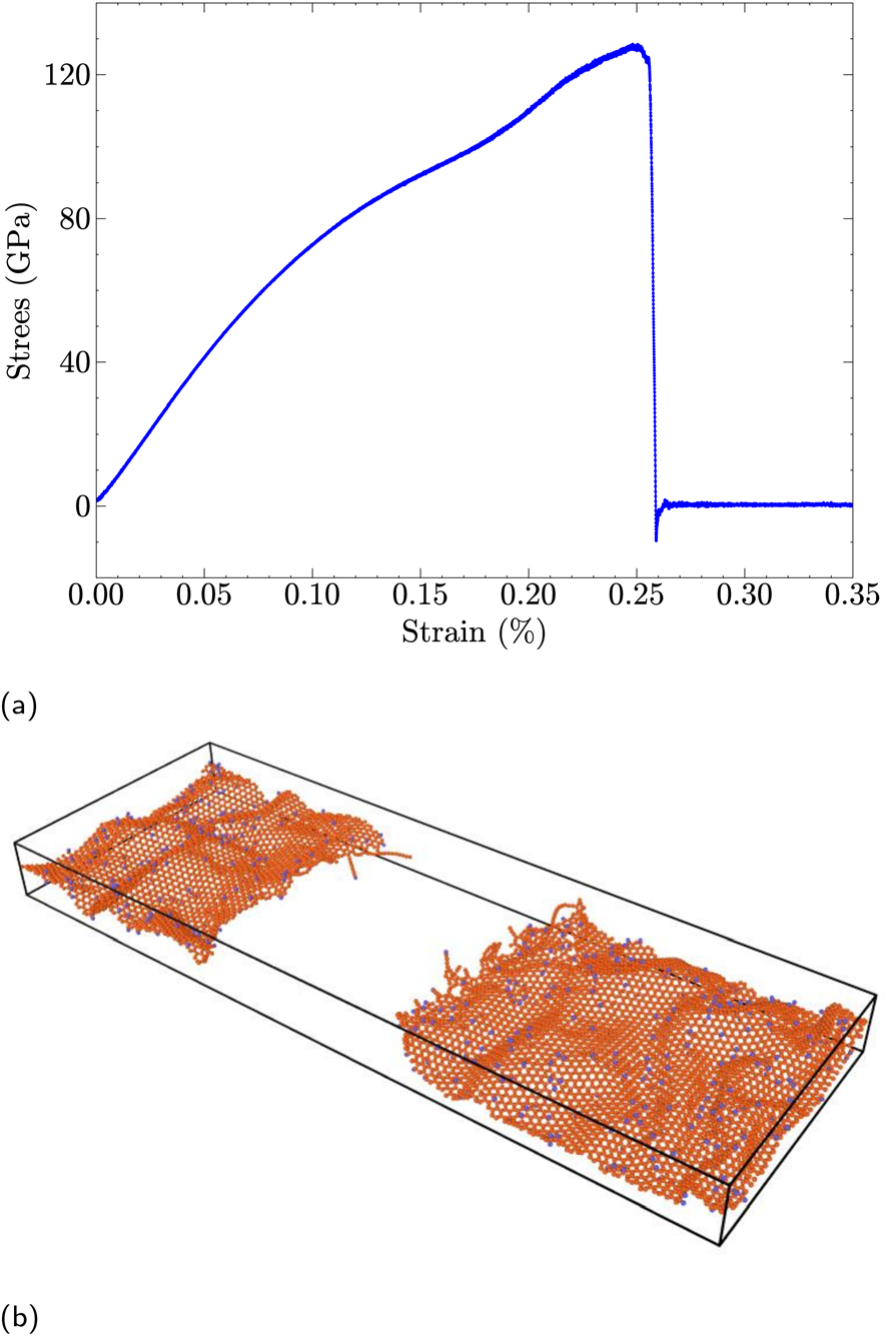
Maximum stress and strain calculation for a hydrogenated graphene sheet with 7% coverage. (a) Stress–strain curve. (b) Fracture visualization.

For mechanical properties, a tensile deformation was imposed along the *x*-axis by employing the “fix deform” functionality in LAMMPS which applies strain by continuously rescaling the simulation box, thereby inducing uniaxial tension in the functionalized graphene sheet; a strain rate of 10^−3^ ps^−1^ was used in our calculations. To calculate total stress in the sheet, the stress per atom^21^ in the system was obtained by1
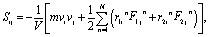
where *i*, *j* indicate in-plane and out-of-plane directions, with *V* and *v* corresponding to sheet volume and velocity, respectively. The first term in eqn (1) corresponds to kinetic energy, while the second term captures the pairwise energy contribution with *N* denoting the number of atom neighbors. Here, *r*_1_ and *r*_2_ pertain to the atom locations, and *F*_1_, *F*_2_ denote the corresponding forces. The nanosheet's net stress is finally calculated by summing the per-atom stresses over all atoms in the system. To validate our LAMMPS setup for these simulations, we first evaluated the mechanical properties of the corresponding pristine monolayer graphene. The computed Young's modulus was 0.99 ± 0.05 TPa, which is in excellent agreement with experimental measurements of 1.0 ± 0.1 TPa.^26^ For the calculation of maximum stress and strain, the stress–strain curve was used and validated. For example, the stress strain curve for a nanosheet with 7% hydrogenated graphene along with the fracture visualization are depicted in Fig. 2. The maximum stress and strain values corresponding to the stationary point before failure are recorded for each case; see Fig. 2a.

**Fig. 3:**
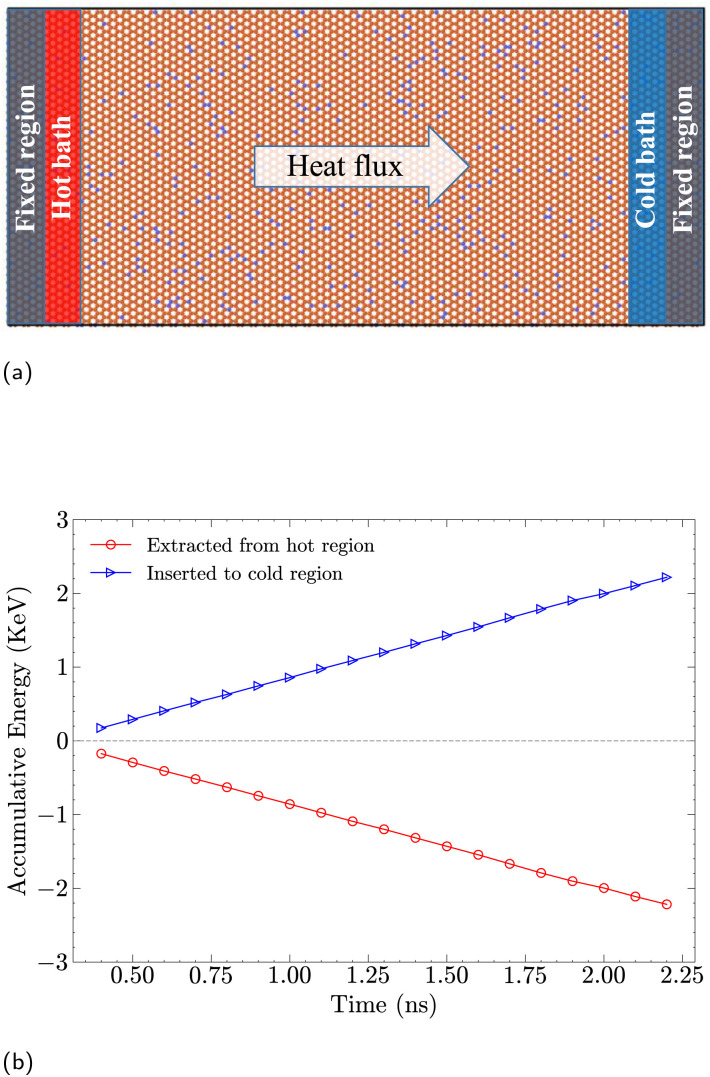
Thermal conductivity: setup and numerical calculations. (a) Schematic representation of the NEMD setup used for thermal conductivity calculations. Fixed regions are applied at both ends of the graphene nanosheet, with adjacent hot and cold baths generating a heat flux across the system. (b) Heat transport for a graphene sheet with 7% hydrogen functionalization between the hot and cold bath.

For the estimation of the nanosheet's thermal conductivity, a different setup is required. Specifically, fixed atoms were considered for two narrow regions at both ends, along the longitudinal (*x*) direction, of the nanosheet to prevent motion during the simulation. Adjacent to these constrained regions, two additional regions were defined as hot and cold baths; see 3a. A temperature difference, (Δ*T* = 40 K), was established by coupling the hot and cold reservoirs to the Nosé–Hoover thermostat (NVT) at 320 and 280 K, respectively, with an NVE ensemble considered for the remainder of the nanosheet. Heat transport calculations were performed by considering a division into slabs with a width of 5 Å along the heat transport direction, and the average temperature for each slab was computed over 5 × 10^5^ steps. Under the applied temperature gradient, the heat flux (energy transferred per unit time and area) reached a steady state after approximately 1 ns, with slight fluctuations around a stable average value. The accumulative energy extracted from the hot reservoir and added to the cold reservoir was then recorded to determine the heat flux; see Fig. 3b. Thermal conductivity was finally calculated using the one-dimensional Fourier's law^24,25^2
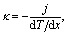
where *j* is the heat flux and d*T*/d*x* is the temperature gradient.

Typical wall-clock time was approximately 4 hours for estimating mechanical properties, with 11 to 25 hours required for estimating thermal conductivity, depending on the complexity of the layout (low to high percentage of functionalization). Simulations were typically performed on one node of Nazarbayev's University Shabyt HPC cluster, with each computing node featuring two AMD EPYC CPUs (32 cores/64 threads per CPU, 64 cores/128 threads per node) and 256 GB of RAM.

### 2.2 Regression models

As previously mentioned, MD simulations pose a significant computational burden. Therefore, we aim to substantially limit the number of required computations by employing surrogate models that can quickly and relatively accurately predict the thermomechanical properties of similar functionalized graphene nanosheets. In this work, we employ several regression models found in the arsenal of ML methods, and assess their performance in predicting the thermomechanical properties of functionalized graphene sheets for two alternative layout encodings. The most successful ones will be ultimately used in the solution of the reverse engineering problem at hand, *i.e.*, determining the functionalization positions for a given set of thermomechanical properties which is practically intractable if only full MD simulations are used. The regression models tested in this work are as follows:

• Ridge regression is a linear regression model with *L*_2_ regularization to prevent overfitting. It assumes a linear relationship between features and target variable. The model estimates the weight vector **w** and the bias term *b* by minimizing the following cost function:3
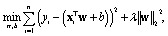
where *λ* > 0 is the regularization parameter that controls the strength of the penalty term in the cost function. The penalty prevents overfitting by shrinking the coefficients, while preserving model interpretability. The value of *λ* is a hyperparameter determined during the training process. Ridge regression was chosen due to its robustness, efficiency, and explainability.

• Support Vector Regression (SVR) is a supervised learning method that extends the principles of Support Vector Machines to regression tasks. Instead of minimizing the squared error, SVR uses an *ε*-insensitive loss function, which allows the model to ignore errors smaller than a specified threshold *ε*. This makes the method robust to small fluctuations in the data while focusing on capturing the overall trend. SVR's hyperparameters are the kernel function, which enables it to model both linear and non-linear relationships, *ε*, which controls the noise level, and a regularization parameter similar to *λ* in Ridge regression. SVR was chosen due to its flexibility, robustness to outliers, and strong generalization performance in high-dimensional feature spaces.

• *K*-Nearest Neighbors (KNN) is a non-parametric regression method that makes predictions based on the *K* nearest neighbors in the training set, as determined by the selected distance metric, *e.g.*, Euclidean distance. The Euclidean distance for 2 samples **x**_*i*_ and **x**_*j*_ with *M* features, is defined as4
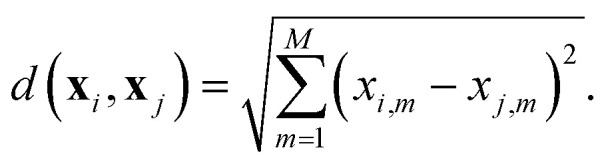


The predicted value for a new sample is computed as the average of the target values of its *K* nearest neighbors. KNN has the advantage of being simple, intuitive, and adaptable to local structures in the data. Its main hyperparameters are the number of neighbors *K*, which controls the balance between model smoothness and sensitivity to noise, and the distance metric, which is used to estimate the nearest neighbors. KNN was chosen due to its simplicity, interpretability, and effectiveness as a baseline method in regression tasks.

• Gaussian Process Regressor (GPR) is a non-parametric, Bayesian regression method that defines a distribution over possible functions that fit the data. Instead of learning explicit weights, GPR assumes that the observed data are generated from a Gaussian process, which is fully specified by a mean function and a covariance function (kernel). For new input, GPR predicts a distribution over possible outputs, providing both a mean prediction and an uncertainty estimate. The main hyperparameters are the choice of kernel and its parameters, such as length scale and variance. GPR has been reported by Chen *et al.*^27^ to achieve >0.95 *R*^2^ score on predicting the thermal conductivity of inorganic materials.

The supervised machine learning process in this study was conducted in three stages: (a) nanosheet layout encoding, (b) training and validation of regression models, and (c) comparison and selection of the best-performing model.

Considering the lack of well-studied vectorization techniques tailored for homogeneous and large structures, such as graphene, it was decided to proceed the application of 2 simple and explainable encoding strategies: Label and Bag-of-Words encoding. This results in 2 sets of models and allows for efficiency comparison of the encoding approaches. Furthermore, these encodings were enhanced by differentiating the functionalization side, *i.e.*, on top or below the nanosheet, so that 3D spatial features of the underlying structure can be extracted. For label encoding, a 2D matrix was constructed with each cell assuming a value from a dictionary that mapped each atom, or functionalization group, to an integer value. Hence, carbon atoms were mapped to the value of 1, whereas carbon-functional group pairs were assigned to the value of 2 or 3 depending on whether the functional group was placed on top or below the graphene plane. Finally, the 2D matrix was vectorized by stacking its rows sequentially and as a result, each distinct functionalized graphene sheet was encoded with a corresponding 8528-dimensional vector with integer components.

For Bag-of-Words encoding, the graphene sheets were encoded as strings of atoms and functional groups, and the resulting strings were tokenized. Afterward, the frequency of each atom and functionalization group were counted and used as features in the feature vector. Such a simple encoding method also allowed us to try and extract other meaningful nonlinear features, such as polynomial features. The degree of polynomial features is a hyperparameter and was tuned for each model separately. To ensure compatibility with certain machine learning models, such as KNN, the features were scaled using the relation 
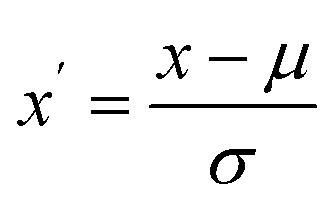
, where *x* is the original feature value, *µ* is the mean of the feature across the training samples, and *σ* is the corresponding standard deviation. This transformation results in features with zero mean and unit variance, improving the comparability of different dimensions in the feature space.

A total number of 1200 MD simulations were performed, as each of the 600 samples had to be separately processed for extracting mechanical properties and thermal conductivity. Subsequently, two datasets (one for hydrogen and one for methyl functionalization) were constructed with each entry comprising the input features (a vector of 8528 integer variables and the output values (thermal conductivity, strain at maximum stress, maximum stress, and Young's modulus). Due to potential noise and discrepancies in property values extraction, the presence of outliers is possible in the data. Therefore, a preprocessing process was applied, pertaining to a careful examination of the simulation results and application of a basic outlier cleaning technique based on Inter Quartile Range (IQR) to each dataset. IQR refers to the absolute distance between the 25th and 75th percentile of the target value distribution: IQR = |*Q*_3_ − *Q*_1_|, where *Q*_1_ and *Q*_3_ denote the first and third quartiles, respectively. A data point *x* is considered an outlier if it satisfies one of the following inequalities: *x* < *Q*_1_ − 1.5 × IQR, *x* > *Q*_3_ + 1.5 × IQR. This rule ensures that extreme deviations from the central distribution are removed, reducing the influence of anomalous points on the regression models. As a result of the outlier cleaning process, 18 data points were removed from the strain training dataset for hydrogen functionalized graphene, and 16 points were removed from the strain training dataset for methyl functionalized graphene. The data for the other target variables was not affected, indicating the absence of outliers.

Subsequently, the hydrogen and methyl datasets were split into training and test sets, with 80% of the data being allocated to the training set, and 20% to the test set. Due to the relatively low number of available samples, tuning of model hyperparameters was performed using exhaustive grid search and 5-Fold Cross-Validation, with the final evaluation being conducted on a separate test set. For Bag-of-Words encoding, given the possibility of nonlinear relations between features and targets, polynomial features was extracted from the data using the PolynomialFeatures preprocessor implementation in scikit-learn library.^40^ The optimal degree of polynomial features is an additional hyperparameter and was tuned along with the remaining hyperparameters of the models.

For tuning the hyperparameters and evaluating the performance of the employed regression models, the following metrics were used. These metrics are well-suited for regression-related machine learning problems:^41^

• Coefficient of determination *R*^2^; see eqn (5),

• Root Mean Square Error (RMSE); see eqn (6),

• normalized Root Mean Square Error (nRMSE); see eqn (7), and

• Mean Absolute Percentage Error (MAPE), see eqn (8).5
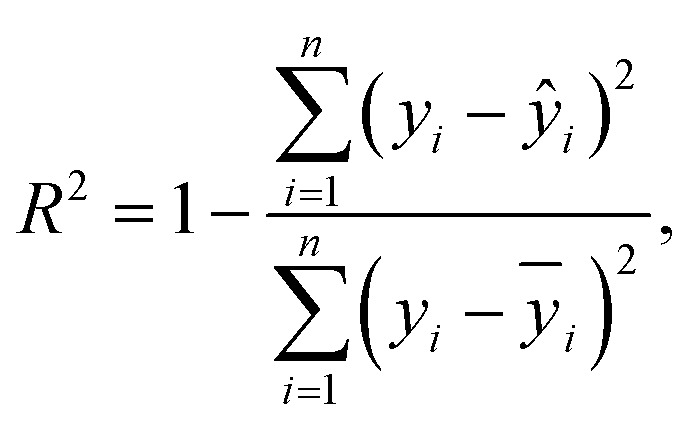
6
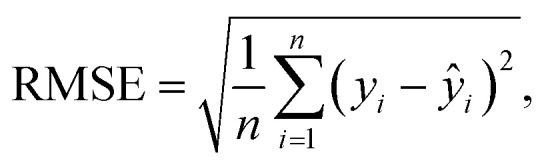
7
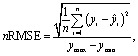
8
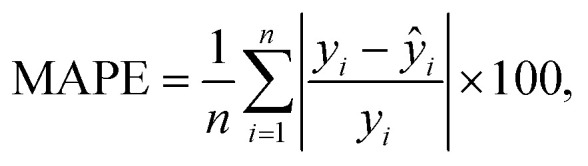
where *ŷ* denotes predicted values, *ȳ* is the mean value, *y*_max_/*y*_min_ correspond to the maximum/minimum value in the dataset, and *n* is the number of employed samples.

The sets of hyperparameters considered in each model were as follows: (a) regularization strength *λ* for Ridge Regression; (b) regularization parameter, error threshold *ε*, and the kernel type (linear, gaussian, or RBF††Radial Basis Function.) for Support Vector Machines, (c) the distance metric (Manhattan or Euclidean) and the number of neighbors *K* for KNN, and (d) the kernel type (RBF, Matérn, or Rational Quadratic), as well as the kernel-specific parameters for GPR. In addition, for Bag-of-Words encoding, the degree of polynomial features was also considered.

### 2.3 Inverse design

The inverse design problem, *i.e.*, the identification of the functionalized graphene sheet layout that exhibits a given set of thermomechanical properties, is addressed through the coupling of the identified regression models with global optimization algorithms, which enable a systematic search of the suitable graphene layouts in the design space. The optimization problem is formulated with label encoding for the design variables, as Bag-of-Words encoding does not allow for the reconstruction of the atomic layout from the optimized vector. Specifically, we follow an approach similar to the one found in Mashhadzadeh *et al.*,^24^ with each component of the design vector, *x*_*i*_, *i* = 1, 2,…, 8528, assuming a value 
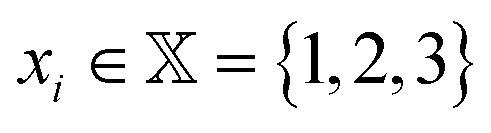
, and therefore our design variable vector 
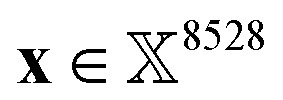
. Hence, each variable corresponds to either a carbon atom or carbon-functional group pair with the additional information of whether the functionalization is on top or below the nanosheet.

**Fig. 4:**
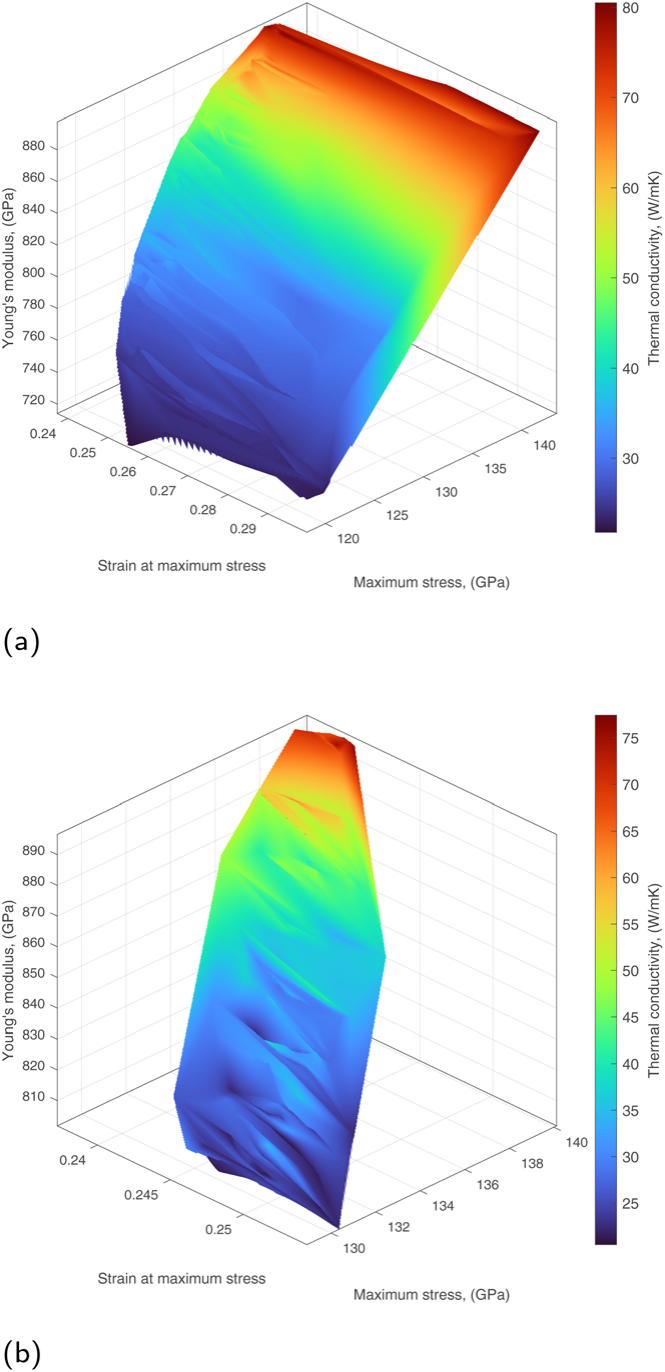
4D hypersurfaces for two datasets. (a) 4D hypersurface for hydrogen-functionalized graphene. (b) 4D hypersurface for methyl-functionalized graphene.

**Fig. 5:**
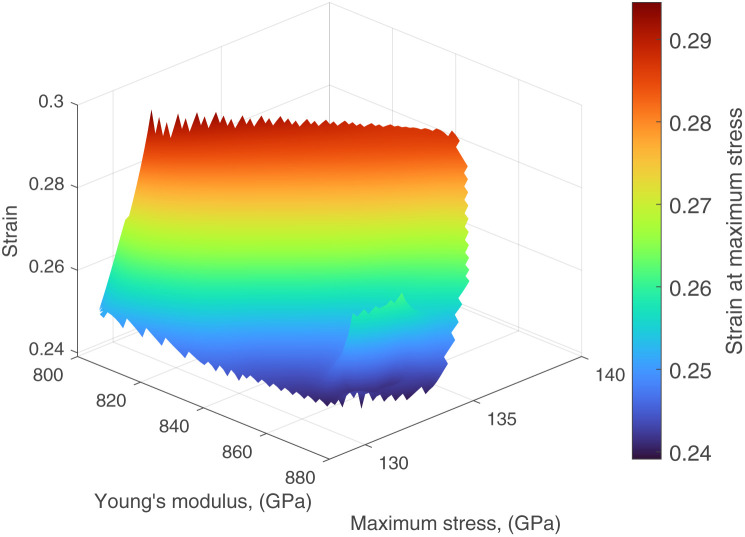
3D slice of the design space hypersurface at *κ* = 50.98.

For benchmarking the capacity of our proposed inverse design approach in identifying nanosheet layouts, a space of feasible thermomechanical properties needs to be determined. This is even more important for surrogate-based inverse design problems^42^ as they do not employ any intrinsic/physical mechanism for discarding invalid property sets. At the same time, we should obviously refrain from using target values that have been used in the training process of the ML models, to avoid trivial solutions. To address these two challenges, we generated a 4D hypersurface, 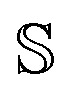, *via* fitting the property values (strain at maximum stress *ε*, maximum stress *σ*, Young's modulus *E*, and thermal conductivity) from the corresponding MD simulations. This enables to pick target values that are potentially feasible while at the same time avoiding 4D points that are in the vicinity of tested nanosheet layouts. The resulting hypersurfaces for hydrogen and methyl functionalization are shown in Fig. 4a and b, respectively. Strain at maximum stress, maximum stress, and Young's modulus correspond to *x*, *y* and *z*-axis respectively, while thermal conductivity variation is represented *via* color mapping. One may directly pick a 4D point from the hypersurfaces or produce a slice when one or more properties need to have specific value(s) with the remaining ones being allowed some variation. Such an example of a 3D slice is shown in Fig. 5 where a fixed value of *κ* = 50.98 is required with the set of remaining feasible properties shown on the plotted surface.

We handle the inverse design problem by formulating and solving the following constrained optimization problem:9
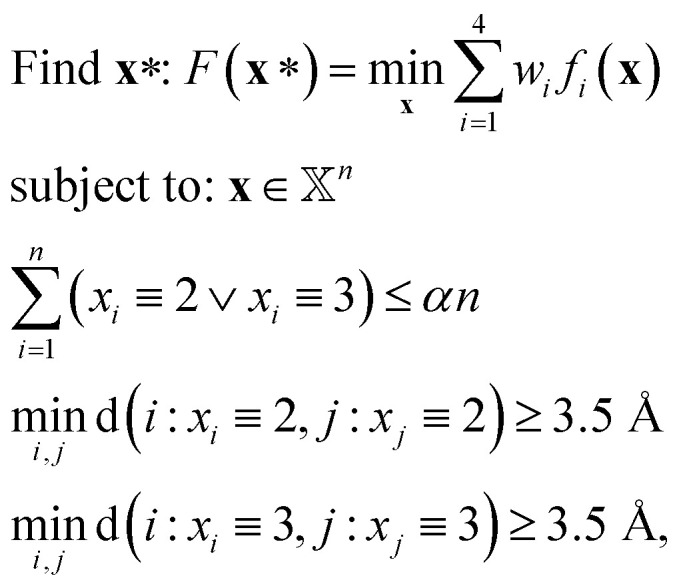
where *n* = 8528, 
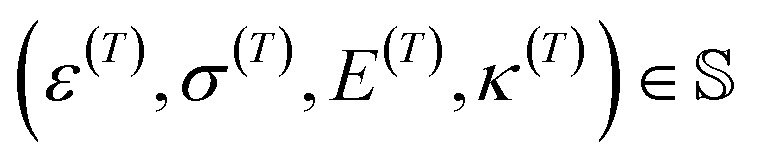
, *w*_*i*_
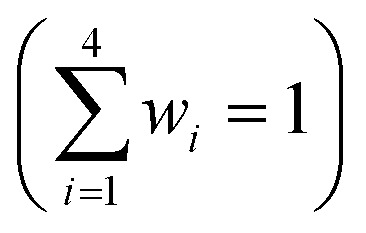
 corresponds to the user-specified weight, *α* is the allowable functionalization percentage, d(·,·) is the distance between two atom positions on the nanosheet, and *f*_*i*_ denote the normalized squared differences between the target, denoted with (*T*), and estimated property values, *i.e.*,10
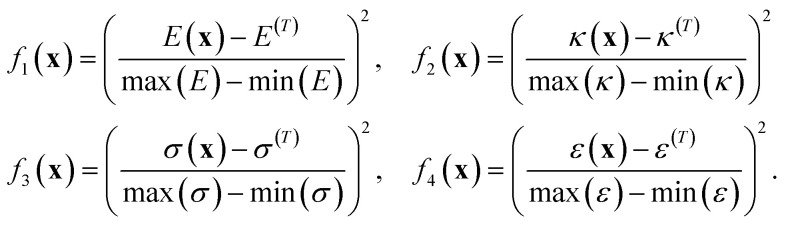


Note that the last two functional constraints in eqn (9) are critical for methyl-functionalization and can be relaxed when only hydrogen atoms are used in functionalization.

The optimization problem in eqn (9) is a constrained integer programming problem that can be solved using MATLAB's implementation‡‡https://www.mathworks.com/products/global-optimization.html of Genetic Algorithms with integer variables, general inequality constraints and simple bounds. Note that the property value predictions in eqn (10) are produced by the corresponding best-performing ML models. Optimization runs were performed on an entry-level workstation with an 8-core AMD Ryzen CPU with 16 GB of RAM. Typical runtime for the ML-enabled optimizations was approximately 1 hour for 20 000 function evaluations, which was sufficient in all tested cased to reach convergence. Finally, following the determination of the optimal layout x*, we validate it by converting back to an appropriate LAMMPS data file and performing the MD simulations as described in Section 2.1. Indicative optimization results along with their validation are presented in Section 3.2 for both hydrogen- and methyl-functionalization cases.

## 3 Results & discussion

**Fig. 6:**
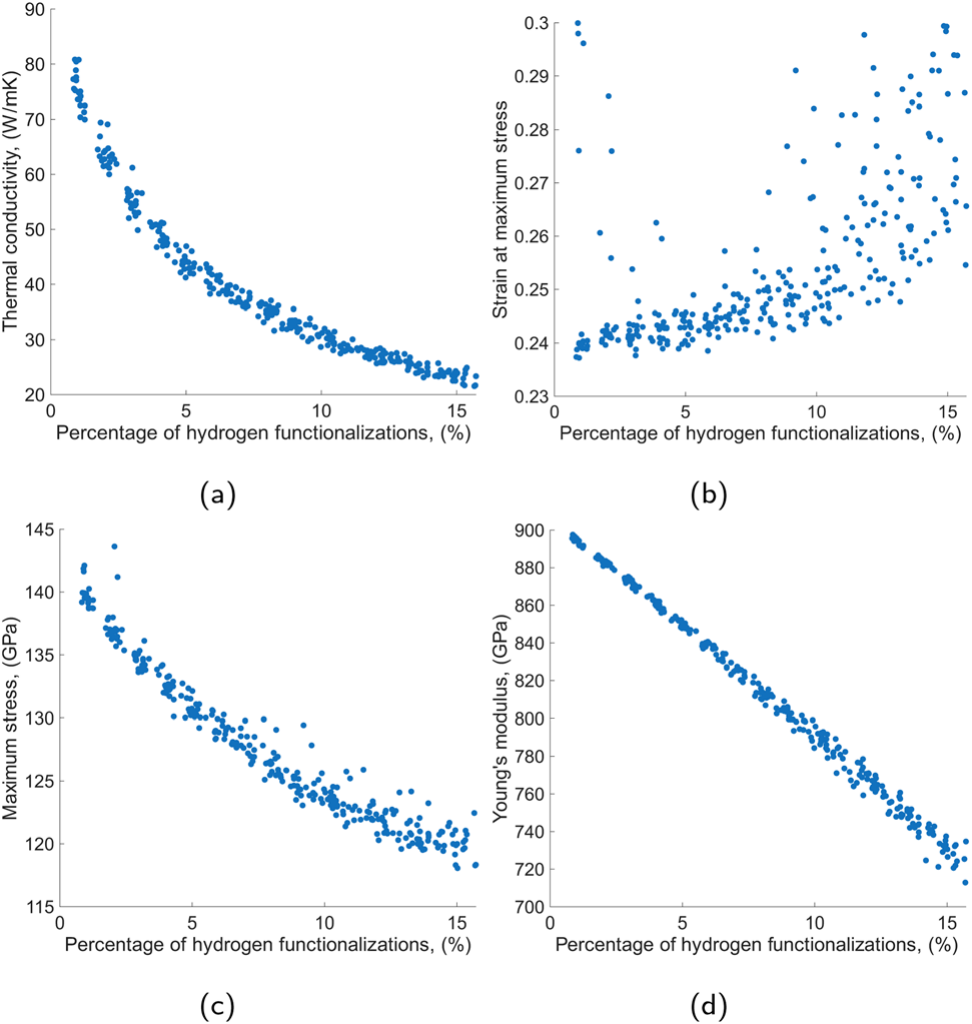
Thermomechanical properties of hydrogen-functionalized graphene with respect to functionalization percentage. (a) Thermal Conductivity. (b) Maximum strain. (c) Maximum stress. (d) Young's modulus.

**Fig. 7:**
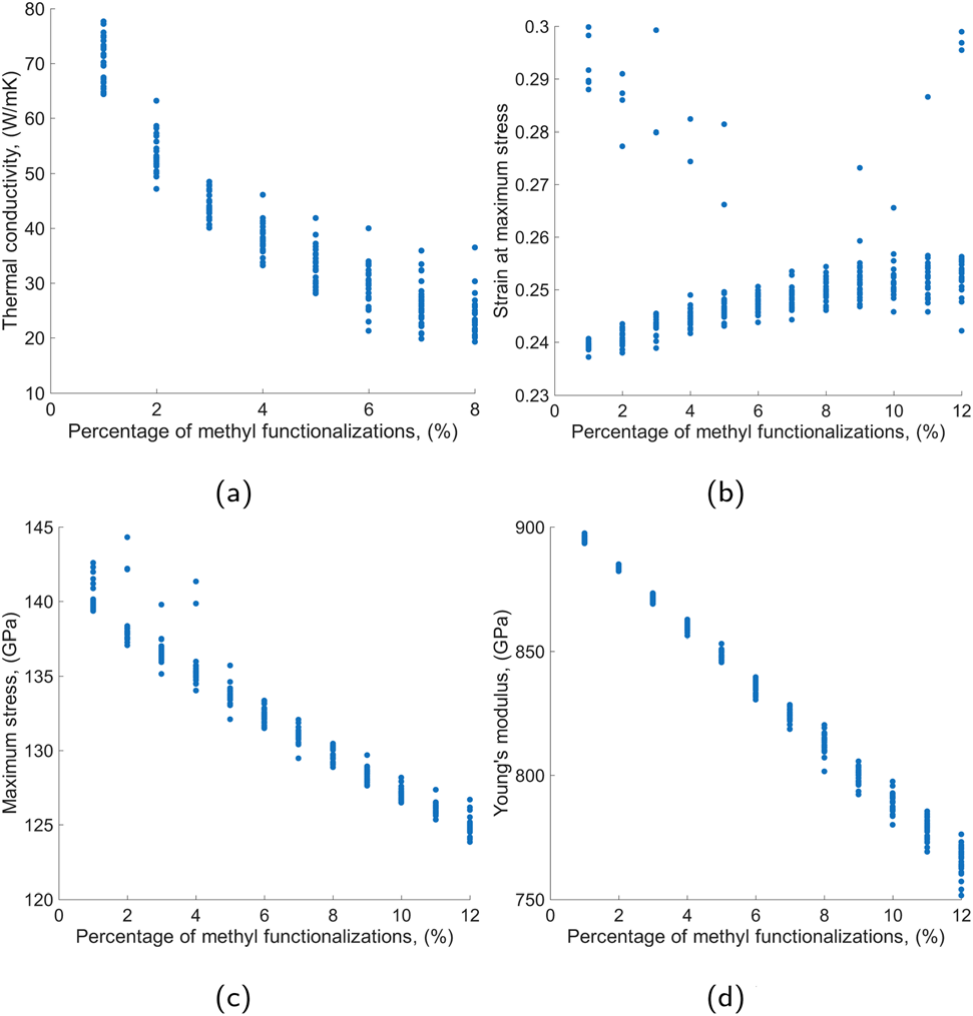
Thermomechanical properties of methyl-functionalized graphene with respect to functionalization percentage. (a) Thermal Conductivity. (b) Maximum strain. (c) Maximum stress. (d) Young's modulus.

**Fig. 8:**
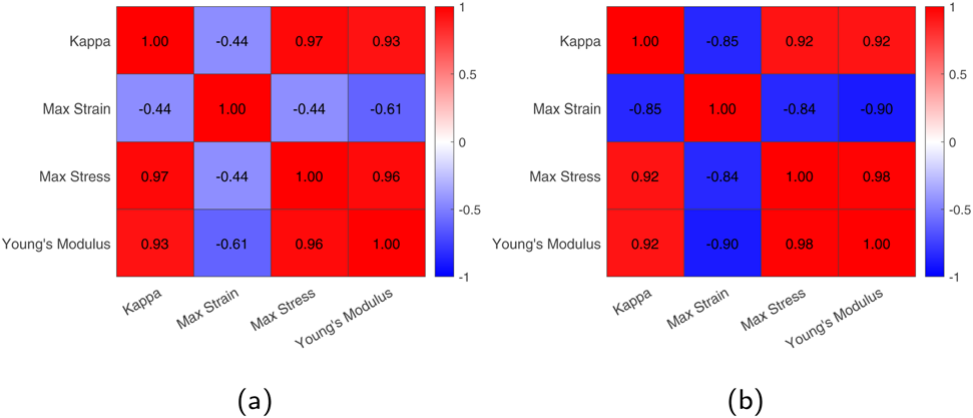
Pearson's correlation matrices. (a) Hydrogen-functionalized graphene. (b) Methyl-functionalized graphene

Before delving into ML modeling results and their use in solving the inverse design problem, we briefly present here a preliminary exploration of the functionalization effects on graphene sheet properties based on the performed simulations. Fig. 6 and 7 visualize these effects by depicting the values of the four properties of interest (thermal conductivity, strain at maximum stress, maximum stress and Young's modulus) against the employed percentages of functionalization, whereas Fig. 8 includes the Pearson's correlation matrices for the same properties, under hydrogen and methyl functionalization.

Apart from the well-studied degradation of graphene properties (thermal conductivity, max stress, Young's modulus) with increased functionalization, these figures reveal a significant property variation, for most cases, at the same level of functionalization. This is highlighted more clearly in Fig. 7 where we chose to depict property values at quantized levels of functionalization. This clearly underlines the importance of functional group placement even when identical percentages are considered, and justifies the consideration of the exact functionalization positions and their distribution in this study. We should also note here that these visualizations include outliers as can be clearly seen in Fig. 6b and 7b. Outliers have been removed from the training sets with the IQR filter technique described previously in Section 2.2.

In more detail, hydrogen-functionalized graphene (Fig. 6) exhibits a practically linear monotonic decrease of Young's modulus with increased functionalization, which, to some extent, applies to maximum stress also. A similar reduction in stiffness of hydrogenated graphene was reported by Dewapriya *et al.*,^10^ and indicates that sp^2^ hybridization is gradually being replaced with sp^3^ hybridization^7^ which weakens the nanosheet. If we ignore some obvious outliers, the strain at maximum stress is close to the reported values for pristine graphene for low functionalization, *i.e.*, around 0.24 which coincides with the experimental study by Lee *et al.*^26^ As functionalization progresses, an increasing upward trend is recorded indicating ductility. A similar behavior is reported in Khoei and Khorrami^43^ for covalent attachment of oxygen and hydroxyl groups to monolayer graphene, and in Xu *et al.*^44^ that reported an increasingly ductile fracture behavior for graphene with an increase in randomly distributed defects coverage. Finally, thermal conductivity exhibits a non-linear reduction relationship with the steepest downward slope occurring at low levels of functionalization (1–5% coverage, or up to 400 functionalized sites, in our case). This is in alignment with behavior report by Chien *et al.*^45^ for thermal conductivity values of hydrogenated graphene. This behavior demonstrates that the initial introduction of defects has the most pronounced effect on thermal phonon transport, while subsequent functionalization has diminishing effects for both hydrogen and methyl functional groups. This sensitivity to defects introduction was also reported by similar studies.^8^ The thermal conductivity reduction is attributed to sp^3^ bonds that act as phonon scatterers and inevitably lead to a reduction in thermal transport. An overall similar picture is drawn for methyl functionalization for the thermomechanical properties of the resulting nanosheets; see Fig. 7. However, in this figure, we can better observe the effect of the functionalization layout as we are depicting property values for multiple layouts at the same functionalization percentage. However, in this case, the layout of methyl functionalization seems to be affecting property values to a larger extent when compared to hydrogen. A relevant study by Pei *et al.*^6^ revealed similar impacts of methyl functionalization on the mechanical properties of graphene. The observed changes in thermomechanical properties for hydrogen and methyl functional groups are summarized in Table 1.

**Table 1:** Effects of hydrogen- and methyl-functionalization on nanosheet's thermomechanical properties

Property	Hydrogen	Methyl
Young's modulus	712.9–897.6 GPa	751.7–897.6 GPa
Maximum stress	118.1–143.6 GPa	123.9–144.3 GPa
Strain at maximum stress	0.24–0.30	0.24–0.26
Thermal conductivity	21.6–80.8 W mK^−1^	19.3–77.7 W mK^−1^

If we finally turn our attention to Pearson's correlation coefficients (see Fig. 8), a strong positive correlation between Young's modulus, maximum stress and thermal conductivity for both hydrogenated and methylated graphene sheets is recorded, while strain at maximum stress is inversely correlated, as we have already mentioned in the discussion above. The overall picture reinforces the assumed correlations and supports the use of surrogate models in the prediction of these properties.

### 3.1 Models' performance

The training and performance comparison of the selected regression models was conducted as previously discussed in Section 2.2, *i.e.*, two encodings over two preprocessed datasets with a separate test set for each case were used for the training phase, while the 4 metrics (*R*^2^, RMSE, nRMSE, and MAPE) in eqn (5)–(8) were employed in the evaluation. The best performing models ranked extremely high with respect to the coefficient of determination, *R*^2^, achieving values larger than 0.97 for Young's modulus, maximum stress, and thermal conductivity in the hydrogen test set. For methyl functionalization, similar performance was achieved for Young's modulus and maximum stress with a slightly lower performance for thermal conductivity, 0.85 ≤ *R*^2^ ≤ 0.91, and strain at maximum stress, 0.75 ≤ *R*^2^ ≤ 0.92. However, the prediction of strain at maximum stress for the hydrogen dataset lagged significantly, only achieving 0.18 ≤ *R*^2^ ≤ 0.47. This can be partially attributed to the remaining outliers in the dataset, which are harder to discern in the hydrogen functionalization case, and significantly affect *R*^2^ values. This is further supported by the significant difference in performance for strain at maximum stress between models using Label encoding and Bag-of-Words encoding. The higher dimensionality of Label-encoded vectors increases the chance of overfitting, which can lead to poorer performance when outliers are present. A quick sensitivity check revealed that by removing 5% of the samples with the largest residuals from the test set, the *R*^2^ score of our best performing models for strain at maximum stress prediction increased from 0.47 to 0.60 for Bag-of-Words encoding, and from 0.18 to 0.30 for Label encoding. These dispersed and hard-to-discern outliers in the hydrogen case are partially stemming from the difficulty of extracting accurate values from the stress–strain diagram for some cases, in which the max stress values oscillate for an extended length of strain values, and thus, although these max stress values are practically identical or even higher, the strain at maximum strain can be affected significantly. While most of the tested graphene sheets experience sudden fracture, some of the tested nanosheets show this post-peak strain behavior. Similar stress–strain curves were demonstrated by Compton *et al.*^46^ and Zhang *et al.*^47^ for multi- and single-layer graphene oxide sheets.

**Fig. 9:**
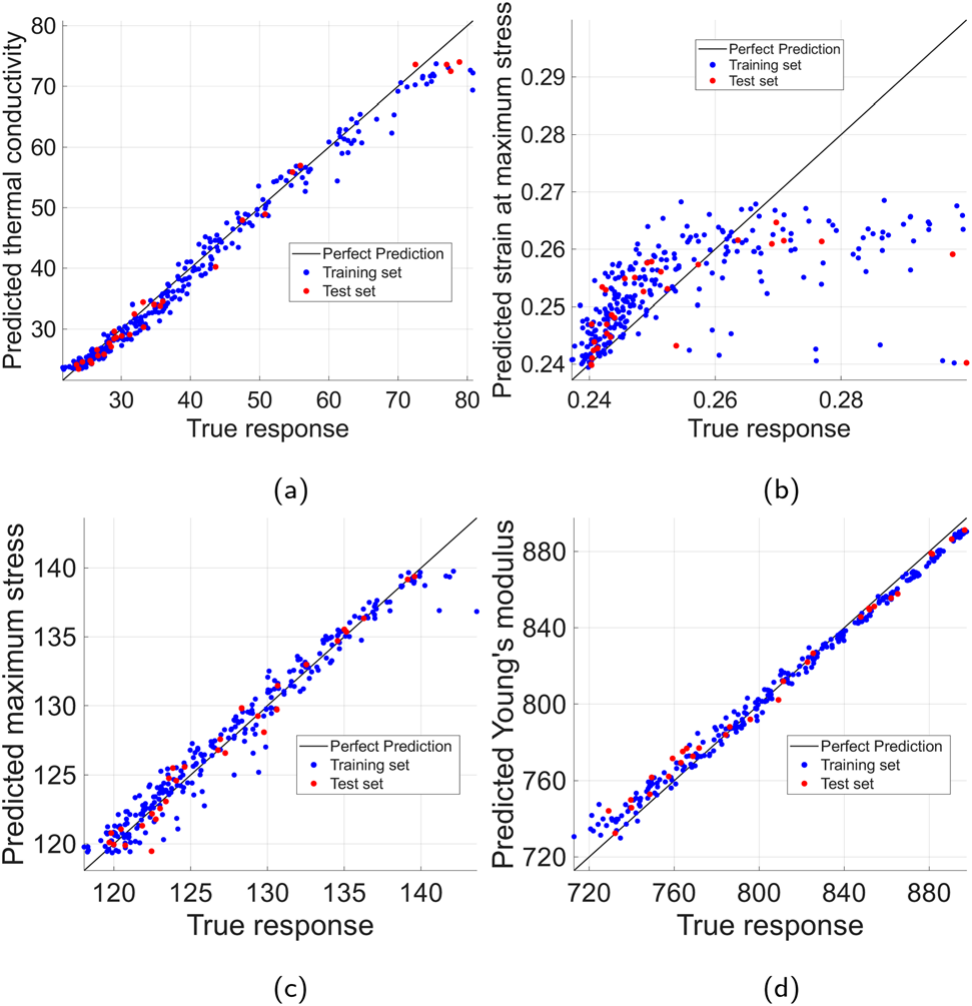
Predicted *vs.* actual property values: training & test sets for hydrogen-functionalized graphene. (a) Thermal conductivity. (b) Strain at maximum stress. (c) Maximum stress. (d) Young's modulus.

**Fig. 10:**
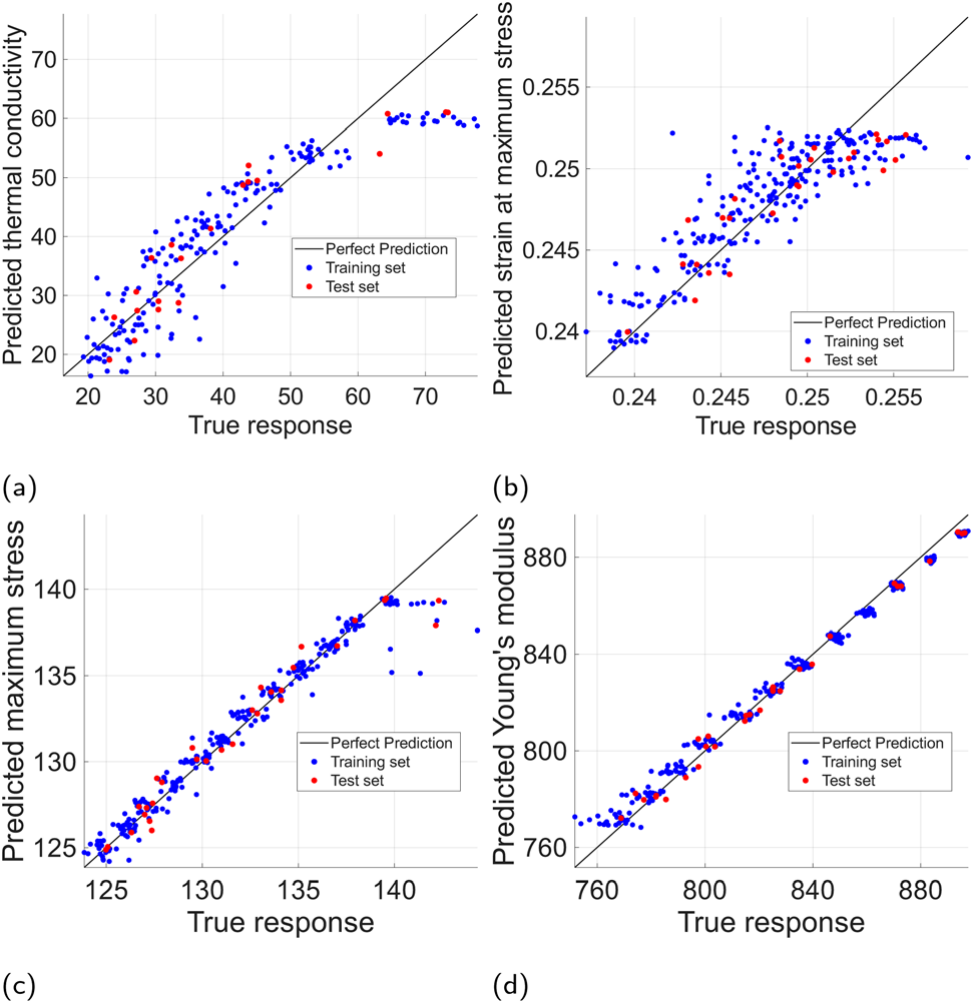
Predicted *vs.* actual property values: training & test sets for methyl-functionalized graphene. (a) Thermal conductivity. (b) Strain at maximum stress. (c) Maximum stress. (d) Young's modulus.

The final results, along with the models' performance with respect to RMSE, nRMSE, and MAPE are summarized in Table 2 for both test sets. The same metrics for the training sets are equivalent or slightly better, which indicates good generalization with no data overfitting, as indicated in Fig. 9 for the hydrogen dataset, and Fig. 10 for the methyl dataset.

**Table 2:** Best-performing regression models with Label and Bag-of-Words encoding for hydrogen and methyl datasets

Functionalization & encoding	Property	Best model	*R* ^2^	RMSE	nRMSE	MAPE
Hydrogen (Label encoding)	Young's modulus	Linear SVR	0.98	5.84 GPa	0.0316	0.5%
	Maximum stress	Gaussian SVR	0.97	0.93 GPa	0.0363	0.5%
	Strain at maximum stress	Gaussian SVR	0.18	0.0145	0.242	3.0%
	Thermal conductivity	Quadratic SVR	0.98	1.95 W mK^−1^	0.0328	3.6%
Hydrogen (Bag-of-Words encoding)	Young's modulus	Ridge	0.99	4.81 GPa	0.0260	0.4%
	Maximum stress	k-NN	0.97	1.09 GPa	0.0426	0.6%
	Strain at maximum stress	k-NN	0.47	0.0093	0.155	3.0%
	Thermal conductivity	Ridge	0.98	1.98 W mK^−1^	0.0334	3.7%
Methyl (Label encoding)	Young's modulus	Linear SVR	0.99	3.76 GPa	0.0258	0.4%
	Maximum stress	Gaussian SVR	0.95	1.16 GPa	0.0567	0.5%
	Strain at maximum stress	Gaussian SVR	0.75	0.00222	0.111	0.7%
	Thermal conductivity	Linear SVR	0.85	6.07 W mK^−1^	0.104	12.7%
Methyl (Bag-of-Words encoding)	Young's modulus	Ridge	0.99	3.73 GPa	0.0256	0.3%
	Maximum stress	k-NN	0.96	1.03 GPa	0.0505	0.5%
	Strain at maximum stress	SVR	0.92	0.0022	0.1	0.7%
	Thermal conductivity	Ridge	0.91	3.95 W mK^−1^	0.0677	7.4%

If we now turn our attention to the remaining metrics, RMSE for Young's modulus and maximum stress are below 1% of property values for both datasets, while for strain at maximum stress the corresponding quantity is similarly low for methyl, and does not exceed 3% for hydrogen. The highest RMSE/MAPE values are observed in thermal conductivity with a value corresponding to around 3.7% for hydrogen and significantly higher, 7–13%, for methyl functionalization. However, the achieved results are still well within the expected fluctuations in MD simulations and the relevant experimental studies in Cao *et al.*^48^ Hyperparameters of the tuned models, that are reported in this section and used in the inverse design can be found in SI. The achieved metric values confirm that the best performing models are sufficiently accurate for employment in property-value prediction for functionalized graphene of both types and consequently, for use in the solution of the inverse problem.

### 3.2 Inverse design results

As we have previously mentioned, for the solution of the inverse design problem we only use the best performing ML models with Label encoding as Bag-of-Words encoding cannot reconstruct the nanosheet layout; see Table 2. The constrained integer programming problem shown in eqn (9) is solved using MATLAB's single-objective Genetic Algorithm (GA) implementation. Multiple GA runs were performed for each of the presented cases to confirm that we have reached a global minimum.

Table 3 summarizes the results of the inverse design for 3 distinct indicative sets of target properties using hydrogen functionalization, while Table 4 presents the corresponding results for 3 additional property sets for methyl functionalization. In both tables, the following values are reported:

**Table 3:** Inverse design results for hydrogen functionalization

Case	Property	Target	Prediction	Validation	Error (prediction-target)	Error (MD-prediction)	Within test RMSE
Case 1	Young's modulus	720.326	720.5378	743.3362	0.0%	3.1%	Yes
	Max stress	119.780	119.6588	120.4692	0.1%	0.7%	Yes
	Strain at max stress	0.277	0.2771	0.25141	0.0%	10.2%	Yes
	Thermal conductivity	23.1473	23.2615	26.996	0.4%	13.8%	Yes
Case 2	Young's modulus	862.871	862.2873	874.6611	0.1%	1.4%	Yes
	Max stress	132.304	132.3983	131.5968	0.1%	0.6%	Yes
	Strain at max stress	0.241	0.2411	0.23753	0.0%	1.5%	Yes
	Thermal conductivity	50.9838	50.9621	49.3276	0.0%	3.3%	Yes
Case 3	Young's modulus	890.140	892.1385	903.879	0.2%	1.3%	Yes
	Max stress	140.025	140.5289	139.8737	0.4%	0.5%	Yes
	Strain at max stress	0.2624	0.2449	0.2400	7.3%	2.0%	No
	Thermal conductivity	76.832	75.1854	73.674	2.2%	2.1%	Yes

**Table 4:** Inverse design results for methyl functionalization

Case	Property	Target	Prediction	Validation	Error (prediction-target)	Error (MD-prediction)	Within test RMSE
Case 1	Young's modulus	892.168	895.6888	823.62	0.4%	8.8%	Yes
	Max stress	139.622	140.0220	140.0797	0.3%	0.0%	Yes
	Strain at max stress	0.24025	0.2399	0.2376	0.1%	1.0%	Yes
	Thermal conductivity	73.7734	72.5505	91.12	1.3%	20.4%	Yes
Case 2	Young's modulus	890.255	888.4488	870.47	0.2%	2.1%	Yes
	Max stress	139.319	139.1904	140.4226	0.1%	0.9%	Yes
	Strain at max stress	0.240	0.2421	0.2377	0.9%	1.9%	Yes
	Thermal conductivity	65.9372	54.6314	84.15	13.4%	35.1%	No
Case 3	Young's modulus	892.806	888.3187	730.91	0.6%	21.5%	No
	Max stress	139.168	139.0302	135.23	0.1%	2.8%	Yes
	Strain at max stress	0.239	0.2405	0.244	0.6%	1.4%	Yes
	Thermal conductivity	69.8553	55.1339	56.83	25.9%	3.0%	No

• Target property values selected on the 4D-hypersurface,

• Predicted property values for the solution of the inverse design problem,

• MD-simulated property values for the solution of the inverse design problem,

• Percentage error between predicted and target property values,

• Percentage error between predicted and MD-estimated property values (*a posteriori* verification with MD simulations of the resulting layout),

• Flag indicating whether the achieved prediction error is within the test-set RMSE for the corresponding surrogate model.

**Fig. 11:**
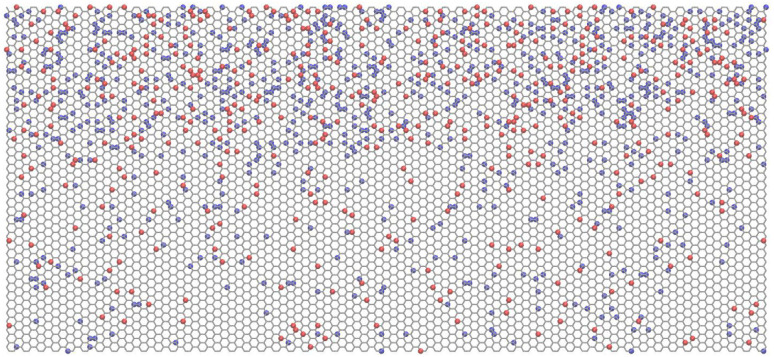
Optimized layout for hydrogen-functionalized graphene case 1; gray hexagonal grid: graphene, orange spheres: H on top, blue spheres: H below.

**Fig. 12:**
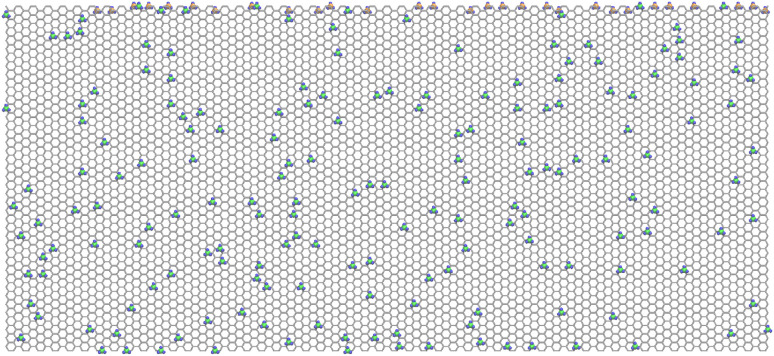
Optimized layout of methyl-functionalized graphene case 3; gray hexagonal grid: graphene, green-blue spheres: methyl on top, orange-blue spheres: methyl below.

Note that among all tested inverse design cases, we selected to include here at least one of the worst performing cases for both datasets (case 1 for hydrogen, and case 3 for methyl) so that we can indicate the maximum expected error in the current level of development of our proposed framework. The resulting nanosheet layouts for these cases are depicted in Fig. 11 and 12, respectively.

If we inspect the obtained results for both cases (Tables 3 and 4), we can generally state that we have succeeded in predicting layouts with properties that are within the expected trained-model deviations, despite having included the worst performers; see the last column in both tables.

For hydrogen functionalization, the smallest observed error is occurring in maximum stress values, with the remaining properties following closely, in most cases. However, we do get some significant deviations (above 10%) between the predicted property values and the *a posteriori* results *via* MD-simulations. This may be due to the relative small training dataset which can be addressed by enriching it with additional MD simulations. Despite the similar overall picture for methyl functionalization cases, the deviations are generally higher when compared to the hydrogen cases. It is worth noting that thermal conductivity exhibited the highest degree of nonlinearity in both datasets, which may have not been sufficiently captured by the trained models due to relative data scarcity. Another interesting observation for this second set, stems from the higher deviations between target and predicted values which did not occur in the hydrogen set of cases. Specifically, a large deviation is observed for cases 2 and 3 where thermal conductivity predictions are far from the target ones.

We consider that apart from the already mentioned issues of non-linearity and data scarcity, this behavior may also be linked, to some extent, to the selection of points on the 4D hypersurface, though inconclusive results have been obtained in our relevant study so far. As both hypersurfaces are constructed on the basis of the current datasets and since regions with limited samples exist, we cannot guarantee the feasibility of all points on the hypersurface. To this end, we should note that for both cases, we have considered target points in regions with higher, equal, and lower local densities, when compared to the normalized 4D point cloud average. For example, although case 3 (see Table 3) corresponds to a target point in a region with a very low density, it achieves significantly better results when compared to case 1 which is located in a region with a relatively higher density. Finally, case 2 is picked in a region with local density significantly above the average, but is only slightly better to case 3. Similar trends are observed in methyl-functionalization cases, *i.e.*, the worst performer is case 3 which corresponds to a region with high-density, whereas cases 1 and 2, showing better results, reside in regions with very low density. Although we can state that picking target points in high-density regions, results in better results for most cases, this is not generally true as we can get equal or better results for target points in extremely sparse regions and rather poor results in relatively dense regions. We consider that these issues can be further investigated in the future by adaptive sampling and/or general enrichment of the available datasets.

Furthermore, if we consider the computational cost of the proposed approach, we achieve at least 10^6^ to 10^7^ orders of magnitude faster predictive performance with respect to pure MD simulations, without sacrificing accuracy. Additionally, we can find valid and optimal (or at least near-optimal) layouts for the inverse problem with 10^5^ to 10^6^ orders of magnitude less computational cost when compared to the employment of MD simulations in the optimization loop.

In summary, and despite the issues mentioned above, we consider that these results indicate that the proposed framework cost-effectively addresses the inverse design problem with sufficient accuracy and therefore, assists engineers in generating valid atomic layouts that can be either directly, or after some relatively minor postprocessing, used to design 2D graphene-based materials with predefined thermomechanical properties.

## 4 Conclusions

In this work, we developed and presented a data-driven framework that enables inverse design optimization of functionalized graphene sheets targeting user specified thermomechanical properties with hydrogen or methyl functional groups. Two datasets, each comprising 300 unique and valid nanosheet layouts with systematically varied functional groups coverage were constructed and evaluated using MD simulations for four thermomechanical properties – Young's modulus, maximum stress, strain at maximum stress, and thermal conductivity. Multiple supervised machine learning models were trained using both Label and Bag-of-Words encoding and compared against four metrics: coefficient of determination (*R*^2^), root mean square error, normalized root mean square error, and mean absolute percentage error. SVR, Ridge regression and KNN models trained with the generated datasets exhibited the best predictive performance of thermomechanical properties for unseen graphene layouts, while showing good generalization (*R*^2^ > 0.9 for the test sets) for three out of four properties, with strain at maximum stress remaining the most challenging property to predict.

Coupling these best-performing surrogate models with a genetic-algorithm-based optimization process enabled the solution of the inverse problem, demonstrating that designing functionalized graphene-based nanosheets with a predefined set of thermomechanical properties is feasible. MD validation of resulting layouts confirmed that in most cases the exhibited thermomechanical properties do not deviate significantly. Mechanical properties seem to generalize better, exhibiting a closer agreement between target, predicted and validated values. However, thermal conductivity is more challenging, especially for methyl functionalization, due to the more pronounced impact of each attached functional group compared to hydrogen case.

Overall, the proposed framework offers an efficient, computationally cheap, scalable and accurate method to design tailored graphene layout for applications requiring specific performance-related properties. These can be attributed to semiconductors, gas separation membranes and water treatment devices.

While the framework shows good results and potential, several challenges remain. First, the dataset of 300 simulations for each functionalization type might not be sufficient for extracting the relationship of features (atomic layout) to the full set of thermomechanical properties. Secondly, this study covers hydrogen and methyl functional groups separately, which limits the applicability of the currently developed framework to a narrow subset of applications. To address these issues, future studies can be performed along the following axes:

• Expansion of datasets to a higher number of simulations and different defect configurations, such as combinations of chemical functionalization and doping.

• Incorporation of graph, convolutional and other neural networks to achieve better generalization of target properties.

• Addressing the curse of dimensionality by finding and testing different encoding strategies that may bring the number of features closer to the number of observed points.

## Author contributions

Ravil Ashirmametov: conceptualization, software, visualization, methodology, investigation, validation, writing – original draft. Alexandr Alpatov: methodology, software, writing – original draft. Farrokh Yousefi: methodology, investigation, writing – original draft. Narges Vafa: methodology, investigation, writing – original draft. Siamac Fazli: methodology, funding acquisition, supervision, writing – review & editing. Konstantinos Kostas: conceptualization, methodology, funding acquisition, supervision, writing – review & editing.

## Conflicts of interest

There are no conflicts to declare.

## Supplementary Material

RA-015-D5RA07646C-s001

## Data Availability

The generated datasets and samples of the LAMMPS scripts are openly available at our GitHub repository (https://github.com/0-daedalus/graphene/tree/main) (README included). Hyperparameters of reported models are provided as SI. Raw simulation outputs are not publicly available and will be shared upon a request. All other data supporting the findings of this study are available within the article. Supplementary information: regarding models' hyperparameters. See DOI: https://doi.org/10.1039/d5ra07646c.
