# Supplementary material for: Determining the structure of functionalized graphene for tailored thermomechanical properties using ML techniques
Source: RSC Adv. 2025 Nov 14;15(52):44423–36. doi: 10.1039/d5ra07646c (PMC12616622; doi:10.1039/d5ra07646c)
Supplement: RA-015-D5RA07646C-s001 [file RA-015-D5RA07646C-s001.pdf]

Supplementary Information (SI) for RSC Advances.
This journal is © The Royal Society of Chemistry 2025

Supporting Information

Determining the structure of functionalized graphene for tailored thermomechanical properties using ML techniques

Ravil Ashirmametov [a*], *Alexandr Alpatov* [b], *Farrokh Yousefi*[a], *Narges Vafa*[a], *Siamac Fazli*[b], *Konstantinos Kostas*[a]

[a]Department of Mechanical and Aerospace Engineering, School of Engineering and Digital Sciences, Nazarbayev University, Astana, Kazakhstan.
[b]Department of Computer Science, School of Engineering and Digital Sciences, Nazarbayev University, Astana, Kazakhstan.
[*]Corresponding author. E-mail: ravil.ashirmametov@nu.edu.kz

1 Models' Hyperparameters

1.1 Hydrogen Functionalization - Label Encoding

- **Target Property: Young's Modulus**
  - Model: SVM
  - Kernel: Linear
  - Box constraint: 287.3936
  - Epsilon: 0.066865
  - Standardization: True
- **Target Property: Maximum Stress**
  - Model: SVM
  - Kernel: Gaussian
  - Kernel Scale: 54.6894
  - Box constraint: 41.1479
  - Epsilon: 0.050525
  - Standardization: False
- **Target Property: Strain at Maximum Stress**
  - Model: SVM
  - Kernel: Quadratic
  - Kernel Scale: 103.0347
  - Box constraint: 0.0137
  - Epsilon: 0.0014
  - Standardization: True
- **Target Property: Kappa**
  - Model: SVM
  - Kernel: Gaussian
  - Kernel Scale: 80.4264
  - Box constraint: 566.4505
  - Epsilon: 0.091427
  - Standardization: True

1.2 Hydrogen Functionalization - Bag-of-Words Encoding

- **Target Property: Young's Modulus**
  - Model: Ridge
  - Alpha: 1.0
  - PolynomialFeatures degree: 2
- **Target Property: Maximum Stress**
  - Model: kNN
  - k: 9
  - PolynomialFeatures degree: 1
- **Target Property: Strain at Maximum Stress**
  - Model: kNN
  - k: 11
  - PolynomialFeatures degree: 1
- **Target Property: Kappa**
  - Model: Ridge
  - Alpha: 0.1
  - PolynomialFeatures degree: 3

1.3 Methyl Functionalization - Label Encoding

- **Target Property: Young's Modulus**
  - Model: SVM
  - Kernel: Linear
  - Box constraint: 3.6977
  - Epsilon: 0.055999
  - Standardization: True
- **Target Property: Maximum Stress**
  - Model: SVM
  - Kernel: Linear
  - Box constraint: 107.0537
  - Epsilon: 0.0064465
  - Standardization: True
- **Target Property: Strain at Maximum Stress**
  - Model: SVM
  - Kernel: Gaussian
  - Kernel Scale: 92.0
  - Standardization: True
- **Target Property: Kappa**
  - Model: SVM
  - Kernel: Linear
  - Box constraint: 6.6544
  - Epsilon: 0.015733
  - Standardization: True

1.4 Methyl Functionalization - Bag-of-Words Encoding

- **Target Property: Young's Modulus**
  - Model: Ridge
  - Alpha: 0.1
  - PolynomialFeatures degree: 3
- **Target Property: Maximum Stress**
  - Model: SVR
  - Kernel: Linear
  - C: 1
  - Epsilon: 0.1
  - PolynomialFeatures degree: 2
- **Target Property: Strain at Maximum Stress**
  - Model: Ridge
  - Alpha: 10
  - PolynomialFeatures degree: 3
- **Target Property: Kappa**
  - Model: SVR
  - Kernel: RBF (Radial Basis Function)
  - C: 10
  - Epsilon: 1
  - PolynomialFeatures degree: 1
